# Recovery of COVID-19 acute respiratory distress syndrome with tocilizumab: successful outcome in two critically ill patients

**DOI:** 10.2217/imt-2020-0154

**Published:** 2020-07-15

**Authors:** Juan David Cala-García, Juan David Sierra-Bretón, Jorge Eduardo Cavelier-Baiz, Álvaro A Faccini-Martínez, Carlos Eduardo Pérez-Díaz

**Affiliations:** ^1^Infectious diseases, Clínica de Marly, Bogotá 110231, Colombia; ^2^Asociación Colombiana de Infectología, Bogotá 110111, Colombia; ^3^Infectious diseases, Hospital La Samaritana, Bogotá 110321, Colombia

**Keywords:** ARDS, C-reactive protein, COVID-19, cytokine storm syndrome, hyperinflammation, IL-6, immunotherapy, monoclonal antibodies, SARS-CoV-2, severe pneumonia, tocilizumab

## Abstract

**Background:** Severe pneumonia and acute respiratory distress syndrome (ARDS) due to COVID-19 is a challenge for nowadays medical practice. Although there is no clarity in the principal mechanism of lung damage and ARDS development, it has been suggested that one of the main reasons of this pathology is the hyperactivation of the immune system, better known as cytokine storm syndrome. Tocilizumab has been proposed to treat COVID-19 severe cases associated to ARDS. **Results & methodology:** Here we present two successful cases of tocilizumab administration in two COVID-19 patients with prior administration of antiviral therapy (hydroxychloroquine, azithromycin, lopinavir and ritonavir) with adequate response and resolution of ARDS, septic shock and severe pneumonia within the first 72 h. **Discussion & conclusion:** This case supports the usage of tocilizumab as an effective therapy in COVID-19 associated cytokine storm syndrome. Further studies should be done in order to assess its effectiveness and security.

In December 2019, global health took a drastic change with the appearance of a novel coronavirus in Wuhan, China. Later, this virus was identified as SARS coronavirus 2 (SARS-CoV-2) and the disease was named coronavirus disease 2019 (COVID-19) [1]. On 1 July 2020, 10,446,353 cases have been confirmed taking over 511,037 lives worldwide, and in Colombia, 102,009 cases have been confirmed with 3470 attributable deaths [2].

COVID-19 has a wide spectrum of disease, ranging from an asymptomatic/mild respiratory infection, to a severe pneumonia with the development of acute respiratory distress syndrome (ARDS) [1]. Although pathophysiology is not completely clear, it has been suggested that in severe cases, a disproportionate immune response might lead to a cytokine storm syndrome (CSS), resulting in damage of the lung parenchyma, pneumonitis, ARDS, viral septic shock and death [3]. This being said, tocilizumab, an IL-6 antagonist, has been proposed for treatment in severe cases [4,5].

Here, we present two Colombian COVID-19 cases successful recovery of severe pneumonia with ARDS after tocilizumab administration in a reference center in Bogotá, Colombia. Administration of tocilizumab was approved as a compassionate off-label protocol under the supervision of the scientific committee of Clínica de Marly medical center. Prior consents were obtained before the administration of the IL-6 antagonist.

## Case reports

### Case A

**Figure 1.**
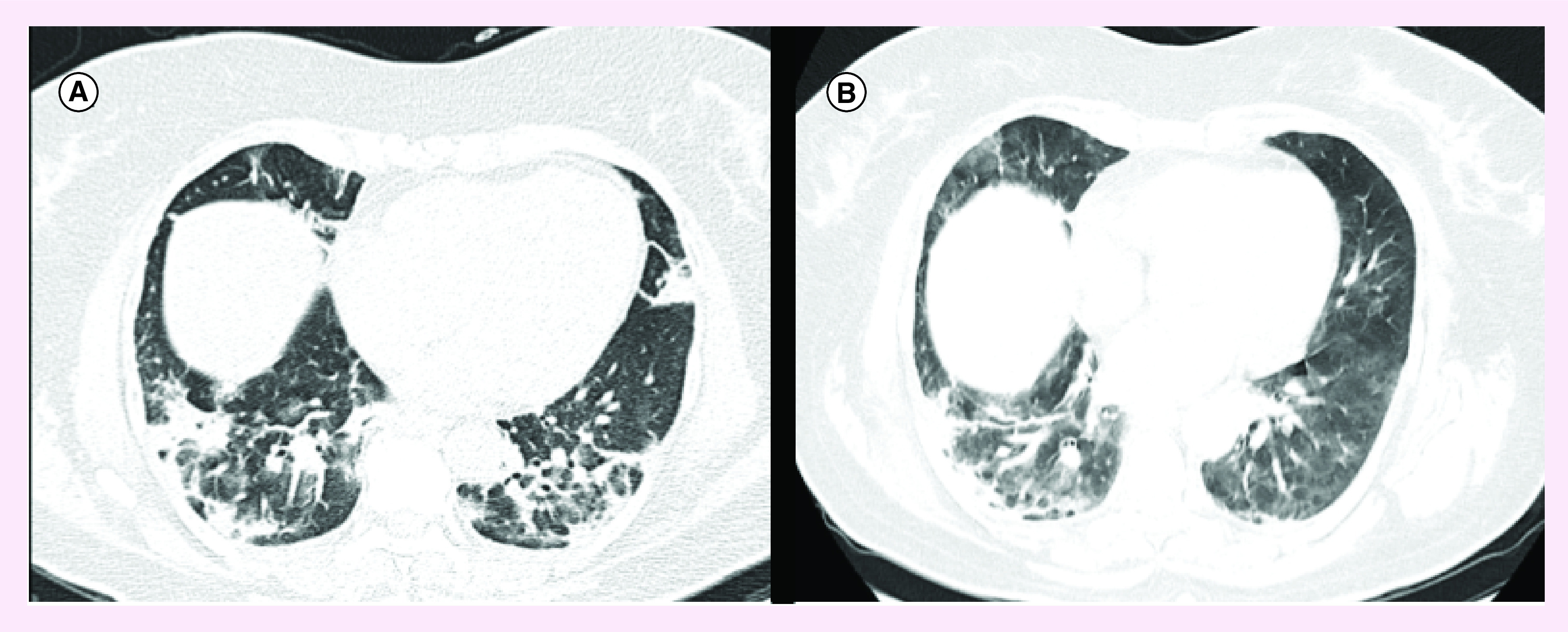
Radiological findings on chest computed tomography from case A. **(A)** On admission (day 0 of hospitalization, day 10 of symptom’s onset). **(B)** On discharge (day 21 of hospitalization, day 31 of symptom’s onset).

A 58-year old female with unknown past medical history who presented with 10 days of dry cough, sore throat, fever and dyspnea. At admission with SaO2 of 85% and costal retractions. Chest computed tomography (CT) evidenced peripheral ground glass opacities (Figure 1A), and initial lab tests were significant for elevated C-reactive protein (CRP) (>9 mg/dl) leukocytosis (20.100 × 10 3/μl), neutrophilia (17.100 × 10 3/μl), elevated lactate dehydrogenase (LDH) (396 U/l) and D-dimer (1.63 μg/ml). Reverse transcriptase-quantiative polymerase chain reaction (RT-qPCR) was performed on an oropharyngeal swap and was found positive for SARS-CoV-2 infection. Treatment with hydroxychloroquine and azithromycin was started, but on day 5 of hospitalization the patient developed septic shock with respiratory failure, with a severe impairment of oxygenation with a PaO2/FiO2 66. The patient was transferred to the ICU in the 7th day of hospitalization; intubation was performed, lopinavir/ritonavir was added as an antiviral treatment and norepinephrine drip was started. During ICU hospitalization, inflammatory markers (D-dimer, LDH, ferritin, CRP) were constantly rising (Table 1), and in the 8th day of hospitalization, 400 mg of tocilizumab intravenous were initiated. After administration, CRP, D-dimer, LDH and ferritin started trending down after 48 h (Table 1), ventilatory support was gradually weaned and extubating was achieved on the 16th day of hospitalization. Patient was discharged on her 21st day of hospitalization (31st day of symptoms onset) with low oxygen support, and control chest CT evidenced diminished ground glass infiltrates but appearance of fibrosis (Figure 1B).

**Table 1.** Laboratory work sheet of case A.

	D1	D3	D5	D8	D10	D12	D14	D18	D21
**WBC (×10^3^ per μl)** Normal values: 4.6–10.2	20.1	10.2	9.77	8.15	6.51	6.36	6.39	6.22	6.38
**PMN (×10^3^ per μl)** Normal values: 1500–8000	17.1	8.41	7.95	5.72	3.91	3.71	3.26	3.60	3.28
**Lymphocytes (×10^3^ per μl)** Normal values: 1000–4000	2.05	1.16	1.34	1.71	2.12	2.25	2.42	1.970	2.52
**Platelets (×10^3^ per μl)** Normal values: 142–424	246	240	276	323	383	414	397	292	402
**Hemoglobin (g/dl)** Normal values: 12–16.8	15.5	12.4	13.5	11.7	13	13	14.3	12.6	16
**LDH (U/l)** Normal values: 120–246		396	335		311	333	339		376
**D Dimer (μg/ml)** Normal values: <0.57		1.63	>5		>5	>5	>5		3.8
**Ferritin (ng/ml)** Normal values: <400			1110		>1000				1050
**Troponin (ng/l)** Normal values: <11			4.1		4.62				<1.5
**C-reactive protein (mg/dl)** Normal values: <1.5	>9			8.4	>9	1.82	0.8		<0.5

Column highlighted shows the day tocilizumab was administered.

D: Day since hospital admission; LDH: Lactate dehydrogenase; PMN: Neutrophil; WBC: White blood cell.

### Case B

**Figure 2.**
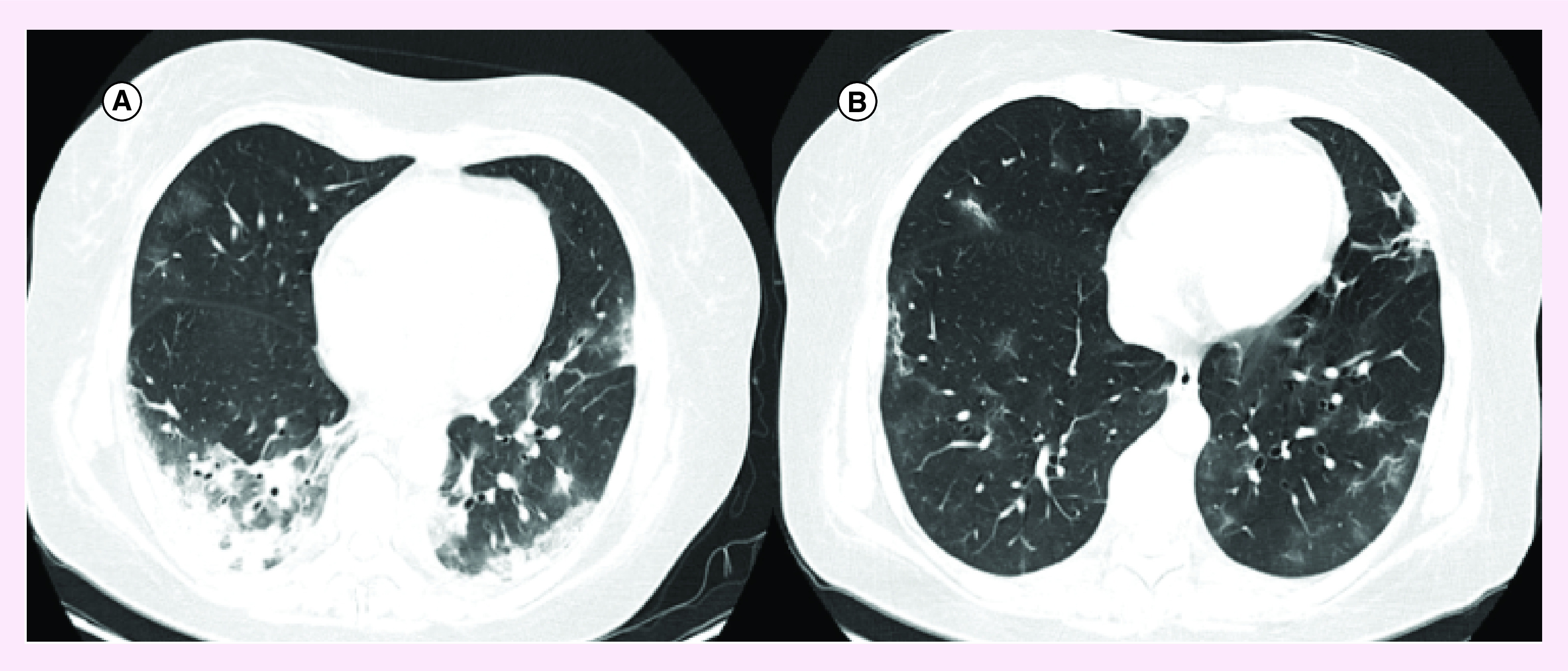
Radiological findings on chest computed tomography from case B. **(A)** On admission (day 0 of hospitalization, day 9 of symptom’s onset). **(B)** On discharge (day 16 of hospitalization, day 25 of symptom’s onset).

A 50-year old female patient with past medical history of hypothyroidism who presented with 9 days of fever, chills, headache and dyspnea. Upon arrival, she was found on a stable clinical condition only with audible rales at physical examination. Chest CT showed ground glass opacities with a peripheral distribution, with greater compromise of bases and areas of crazy paving (Figure 2A), and initial lab tests showed lymphopenia (1010 × 10 3/μl), and lightly elevated LDH (343 U/l), D-dimer (0.88 μg/ml) and CRP (5.09 mg/dl) (Table 2). SARS-CoV-2 RT-qPCR was performed on an oropharyngeal swab which was positive. Hydroxychloroquine/azithromycin was started. However, during hospitalization, her condition worsened with new onset of dyspnea, fever spikes and diarrhea. Lopinavir/ritonavir was started but pneumonia kept progressing with compromise of oxygenation evidenced by lowering of PaO2/FiO2. The patient was transferred to the ICU in the 4th day of hospitalization to provide invasive mechanical ventilation and norepinephrine administration. LDH, ferritin, D-dimer and CRP kept rising (Table 2), and 600 mg of tocilizumab intravenous was administrated on the 7th day of hospitalization. Inflammatory markers kept rising on the first 2 days after tocilizumab, with later regression of D dimer, ferritin, CRP and LDH. Patient improved her clinical condition and was taken out of the ICU on the 12th day of hospitalization and was discharged on the 16th day of hospitalization with low oxygen support (25th day of symptoms onset). Control chest CT showed regression of ground glass opacities with significant improvement (Figure 2B).

**Table 1.** Laboratory work sheet of case B.

	D1	D3	D5	D7	D9	D11	D13	D15
**WBC (×10^3^ per μl)** Normal values: 4.6–10.2	4.66	5.67	7.59	8.14	8.63	7.94	8.63	7.67
**PMN (×10^3^ per μl)** Normal values: 1500–8000	3.20	4.20	6.43	5.79	6.02	6.32	6.23	4.51
**Lymphocytes (×10^3^ per μl)** Normal values: 1000–4000	1.01	1.00	0.718	1.41	1.60	0.91	1.24	1.87
**Platelets (×10^3^ per μl)** Normal values: 142–424)	207	210	207	374	366	423	418	415
**Hemoglobin (g/dl)** Normal values: 12–16.8	14.7	12	9.8	12.5	12.7	11.8	12.5	12.8
**LDH (U/l)** Normal values: 120–246	343	316	364	395	505	328	351	327
**D Dimer (μg/ml)** Normal values: <0.57	0.88	1.18	0.88	1.19	2.7	3.66	2.6	1.78
**Ferritin (ng/ml)** Normal values: <400	183	170	226	370	496	572	598	452
**Troponin (ng/l)** Normal values: <11		3.7	17.1	5.5	8.6	1.55	<1–5	<1.5
**C-reactive Protein (mg/dl)** Normal values: <1.5		5.09	8.9	>9	>9	3.25	0.79	<0.5

Column highlighted shows the day tocilizumab was administered.

D: Day since hospital admission; LDH: Lactate dehydrogenase; PMN: Neutrophil; WBC: White blood cell.

## Discussion

We present two successful cases of tocilizumab treatment in Colombian patients with COVID-19 severe pneumonia and ARDS. Both patients, presumably developed CSS which resolved after receiving IL-6 antagonist. Fortunately, none of our patients developed any adverse reaction related to tocilizumab.

Although it is not entirely clear how SARS-CoV-2 causes severe pneumonia, septic shock, ARDS or even death, it has been suggested that one of the main causes that affects patients is the immune response, specifically, the development of CSS [3,6]. It has been established that SARS-CoV-2 targets cells expressing angiotensin-converting enzyme-2 receptor [7,8], which explains why this virus generates a lower respiratory tract infection, but still, it is not clear what marks susceptibility to develop a severe infection. Patients with severe infections have shown to have high inflammatory markers such as IL-6, CRP, D dimer, LDH and lymphopenia [1,5,9]. Initially, the immune system will start an innate response, recognizing pathogen-associated molecular patterns (PAMPs) and damage-associated molecular patterns (DAMPs), which will increase IFN-1 production and then, generating an activation of LT CD4^+^ Th1, CD8^+^ and LB cells [7]. In cases in which there is an overexpression of the immune system, overproduction of proinflammatory cytokines such as IL-1, IL-6, IL-8, TNF-α will disrupt the integrity of blood vessels, and will create vascular leakage [10], eventually causing alveolar flooding [11]. This correlates well with findings on autopsies on COVID-19 deceased patients which evidences diffuse alveolar damage, proteinaceous exudates, vascular congestion, mononuclear inflammatory infiltrates, mostly lymphocytes and multinucleated giant cells [12].

Tocilizumab is an IL-6R monoclonal antibody, which is currently used mainly for the management of rheumatoid arthritis, and nowadays, with an increase of its use for pathologies associated with CSS such as hemophagocytic lymphohistiocytosis, macrophage activation syndrome, or chimeric antigen receptor (CAR)-T cell therapy adverse reactions [10,13]. Since one of the principal reasons of developing acute lung injury and ARDS in COVID-19 pneumonia is the overexpression of the immune system, blocking IL-6 would stop the activation of innate immune cells (like neutrophils and macrophages) [6], which eventually leads to the development of CSS and therefore, have a decrease in mortality in critically ill patients [14,15].

Currently, there is a Phase III trial (ChiCTR2000029765) in China studying the efficacy and safety of tocilizumab as a treatment for SARS-CoV-2 pneumonia [16], and there has been reported successful usage of tocilizumab lowering inflammatory markers, increasing lymphocyte count and improving ventilatory patters, as well as improvement of radiologic pattern [4,5,17], findings that correlate well with our described patients.

Although there is good evidence of tocilizumab as a target therapy in critically ill COVID-19 cases, there have also been reported failures in tocilizumab administration as treatment of severe illness from COVID-19, in which progression to hemophagocytic lymphohistiocytosis was evidenced, and even development of severe complications such as viral myocarditis was shown [18]. Radbel *et al.* hypothesized that the development of viral myocarditis could be attributed to the decreased lymphocyte maturation due to the IL-6 antagonism [18], raising the concern of immunotherapy in COVID-19 cytokine storm syndrome (CSS). Fortunately, none of our patients developed any complications of immunotherapy, instead they evolved to a complete resolution of ARDS, septic shock and severe pneumonia within the first 72 h.

Although tocilizumab seems like a good option for CSS in COVID-19 pneumonia [13,19], special consideration should be given to its side effects, principally immunosuppression. For tropical countries such as Colombia, we consider screening of TB with QuantiFERON TB before giving tocilizumab in order to prevent possible exacerbations [20].

To summarize, we report the usage of tocilizumab in two critically ill patients with COVID-19 pneumonia, with prior use of antiviral therapy (hydroxychloroquine, azithromycin, lopinavir and ritonavir) with adequate response and resolution of ARDS, septic shock and severe pneumonia within the first 72 h, supporting it’s usage as a possible treatment in a pandemic urging for safe and effective solutions in the setting of critically ill patients. Further randomized controlled clinical trials should be done in order to assess the real effectivity and safety of tocilizumab treatment in COVID-19.

There are still many things we have to learn in order to treat COVID-19 correctly. One of the strongest hypotheses that has been suggested in the academic world is the association of a disproportionate immune response with a severe viral pneumonia and the development of ARDS. Impacting in the inflammatory response, and, in this case, stopping the development of the CSS in COVID-19 might be the correct direction to impact on morbidity and mortality in COVID-19 severe cases.

**Executive summary**

- Cytokine storm syndrome is a reality in severe cases of COVID-19.
- Tocilizumab can be a suitable and effective therapy for severe cases of COVID-19 associated with acute respiratory distress syndrome.
- In our report, tocilizumab was associated with a resolution of acute respiratory distress syndrome in the first 72 h after it’s administration.
- Even though tocilizumab was an effective therapy in our patients, special consideration has to be established for the associated immunosuppression.
- For tropical countries such as Colombia, we consider screening of TB with QuantiFERON TB before giving tocilizumab in order to prevent possible exacerbations.
- Further research should be done in order to establish tocilizumab’s effectiveness and safety.
